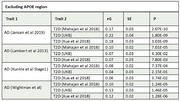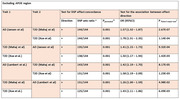# Assessing genetic overlap of Alzheimer’s disease with type 2 diabetes

**DOI:** 10.1002/alz.090165

**Published:** 2025-01-03

**Authors:** Emmanuel O Adewuyi, Tenielle Porter, Giuseppe Verdile, Simon M. Laws

**Affiliations:** ^1^ Centre for Precision Health, Edith Cowan University, Joondalup, Western Australia Australia; ^2^ Collaborative Genomics and Translation Group, School of Medical and Health Sciences, Edith Cowan University, Joondalup, Western Australia Australia; ^3^ Curtin Medical School, Curtin University, Bentley, Western Australia Australia; ^4^ Curtin Health Innovation Research Institute, Curtin University, Bentley, Western Australia Australia

## Abstract

**Background:**

Whilst numerous studies have explored the relationship between Alzheimer’s disease (AD) and diabetes, there remains significant conflicting evidence as to their relationship. Some studies suggest an increased likelihood of developing AD in individuals with diabetes, especially type 2 diabetes (T2D) and that both diseases share pathological features. In contrast, other studies indicate that T2D is more aligned with vascular cognitive impairment and dementia and associated cerebrovascular/white matter pathology. Moreover, there is evidence showing no significant genetic correlation between the two disorders. Understanding the genetic relationship between these potentially comorbid conditions could offer insights into AD’s poorly understood biological mechanisms. This study used two complementary methods to evaluate the genetic relationship between AD and T2D. We hypothesise that both disorders are, to an extent, genetically correlated.

**Method:**

We performed an extensive analysis of large‐scale genome‐wide association summary data using the ‘linkage disequilibrium score regression’ analysis (for global correlation) and ‘single nucleotide polymorphism (SNP) effect concordance analysis’ (SECA, for genetic overlap) methods. We performed several analyses testing our findings’ potential (partial) replications, using several GWAS data for AD (with and without the *APOE* region) and T2D.

**Result:**

We found a highly significant positive genome‐wide (global) genetic correlation between AD (clinically diagnosed with proxy cases) and all T2D GWAS assessed, with or without the *APOE* region. We largely replicated the positive and significant results using clinically diagnosed AD GWAS. Utilising SECA, we found robust SNP overlap and strong effect concordance with low permutation p‐value and significant Fisher’s exact test—underscoring the strong association between SNP sets (and association in effect direction) for both disorders. Our SECA results were consistently significant irrespective of whether AD or T2D was dataset 1 or dataset 2. We replicated SECA’s results across clinically diagnosed AD and other T2D GWAS data (with or without the *APOE* region included).

**Conclusion:**

Our analyses reveal a notable genetic correlation and overlap between AD and T2D, indicating shared genetic factors (shared genetic foundation) and biological pathways influencing the risk or susceptibility to both conditions.